# Calomel: Its Properties, &c.

**Published:** 1889-02

**Authors:** H. Christopher

**Affiliations:** St. Joseph, Mo.


### Southern Medical Record.

A MONTHLY JOURNAL OF PRACTICAL MEDICINE.

All communications must be addressed to R. C. WORD, Managing Editor.

"*Quicquid Præcipies Esto Brevis.*"

VOL. XIX ATLANTA, GA., FEBRUARY, 1889. NO. 2

### ORIGINAL AND SELECTED ARTICLES.

### CALOMEL: ITS PROPERTIES AND INDICATIONS.

BY H. CHRISTOPHER, M. D., ST. JOSEPH, MO.

We recognize morbid action by the manifestations it makes of itself, through the effects it produces on tissues, the disturbance it occasions in the functions of organs, and the symptoms of various kinds of a less definite character to which it gives rise. Sometimes it happens that these indices of disease are so confused, indistinct, or indefinite, or their meaning so ambiguous, that the physician finds his way obstructed by doubts, and his mind so embarrassed, that he discovers the fact that all he can do is to meet the symptoms as they appear, and wait for more definite indications of treatment, until the symptoms are such as to enable him to adopt a more rational course of procedure. But where he has from the first a pretty clear conception of the nature and trend of the morbid action he has to treat, and at the same time knows that he has the means of arresting and terminating the disease in hand, he has that knowledge which leads to a rational course of treatment. This knowledge embraces the nature and trend of the disease in hand, and of the properties and action of the remedies indicated by the nature of the morbid action. In illustration of the general principles, I submit a few reflections on the properties of, and indication of use for, the chief salt of mercury, the mild or mercurous chloride.

The leading properties of this remedy, as pointed out by my own experience may be stated in order as 1. *a cholagogue*, in which action it restores the deranged function of the liver, and contributes to the arrest of inflammatory action and fever, by stimulating its function and thereby causing a more abundant flow of its secretion; 2. as *an absorbent* by which is meant its power to effect the absorption of inflammatory products; 3. as *counteracting or preventing the injurious effects of such inflammatory products* by altering or destroying the nature of the morbid action; 4. as *possessing the power to allay irritation and subdue morbid action of an inflammatory character*, wherever it may occur in the body, from whatever disease arise or whatever attend; 5. as *possessing the power to restore altered or suppressed functions of organs in general*; and 6. as *an anodyne.* and—

1st. As a cholagogue, an exciter of bile flow, or increased activity of the function of the liver. That mercury acts on the liver there is not the shadow of a doubt. It matters not what experiments on *dogs* may show; the evidence that it acts on the liver of man is just as convincing as that ptyalism is a proof that it acts on the the salivary glands. This fact no one, I presume, for a moment questions. The character of the stools following its administration, and its effect on the pulse place the fact beyond a doubt.

2. As an absorbent. A case in illustration will show what this action is, and prove that the claim is unquestionable. In the winter of '49-'50, a negro man was struck on the eye by a snowball thrown by a boy. The man was strong and robust, and in good health at the time of the injury. The pain was very severe which was attended by considerable lachrymation. There was but a slight redness of the conjunctiva; but fever soon set in, and was present when I first saw him. A purgative of calomel with comp. ex. coloc. was given, and the eye shaded and the man kept in the house. The purgative abated the fever, and reduced the pain considerably, but within forty-eight hours after the injury, a cloudy film appeared over the lens. The effusion of coagulable lymph had followed the injury, and the cloud showed that it was becoming oɹganized, threatening the loss of the eye by cataract. Leeches were at once applied around the orbit, and calomel in two grain doses with a half grain of opium was given every two hours, with the view of absorbing the yet soft lymph. It was continued until the breath showed the contitutional effect of mercury. When this took place, the cloudy film began to disappear, and within forty-eight hours the lens was perfectly clear again. The calomel, as I take it, produced absorption of the coagulable lymph, and thus showed its absorbent power. This was a case within sight, and affords a rational ground for believing that a like action may go on beyond sight. If such a thing should occur in our own persons, we would hold the remedy in the highest estimation.

3. As mercury is an absorbent, it follows that it also possesses the property of controlling inflammatory action, resulting in preventing or arresting the effusion of organizable lymph, and of destroying its plasticity after or before its effusion. Under such circumstances, we have no membranous adhesions as the result of high inflammatory action, as in the inflammation of serous membranes. And yet in cases of an aplastic condition of the blood, as when incised wounds do not heal by the first intention, mercury, in the form of the mild chloride, will correct or alter this condition, so that incised wounds will readily heal by the first intention. This fact is so familiar that it needs only to be restated.

4. The influence of mercury on morbid irritation, or a sub-inflammatory condition of a tissue or organ is another property that this remedy possesses. Whether the irritation be limited to the affected organ, or arises by metastasis or likeness of structure in another near or distant organ, it may be relied on with no little confidence, for arresting and subduing such irritation. It is, consequently, indicated in such cases. An instance occurs to my mind at this time. In 1852, I was treating a child aged near a year, in the summer, when the temperature, both day and night, was disagreably high, in an attack of bilious dysentery, attended by the usual symptoms. After treating the child for several days, chiefly with calomel at intervals, the case seemed to come to a halt, and I began to fear cerebral irritation, which is so often seen in cholera infantum. Not being satisfied to proceed farther with the treatment, I consulted Dr. M. L. Linton. I was then in St. Louis. Dr. Linton I esteemed as possessing the best mind in medicine that I ever met. I gave him the case in detail, and asked his advice. He endorsed the treatment. This was very gratifying, but not satisfying, and so I asked *why* he endorsed the continued treatment in the smaller doses. I was then giving calomel and was fearing cerebral irritation. His reply impressed my mind indelibly: "I would give the calomel, let the irritation be where it may." Such was his belief as respects the influence of mercury over inflammatory irritation, and my own experience since has confirmed the correctness of the treatment.

5. In non-inflammatory affections where there exists only a disturbance of function, in a greater or less degree, mercury has the power of restoring the function of the organ suffering, and particularly those of the digestive system. Chief among these is the liver; at least, it is in the disturbed function of this organ that we can the better perceive the action of mercury over these organs, since a functional derangement of the liver shows itself in the pulse particularly.

6. And lastly, as an anodyne. This may excite a smile; but the *genial* smile can be seen on the face of the patient who has suffered from an attack of acute muscular rheumatism, which opium has failed to relieve, as it always does. Does any one know *how* an anodyne acts, how it relieves pain? Can any one explain how quinine cures an acute attack of neuralgia? Nevertheless it cures, and better than opium. In this is it not an anodyne? Now, in acute rheumatism of a muscle or muscles, calomel in large doses relieves the patient of pain most effectually, and very frequently very quickly. In May, 1866, I was called to see a gentleman 6 A. M., who was suffering from a severe attack of rheumatism of the muscles of the left illiac region. He had had a like attack before, and was given opium for it, as he informed me. But the attack continued three days, and no wonder as opium was relied on. I assured him that he would not suffer so long this time. I gave him 5 grains of calomel, a pill every two hours, the first dose being taken at 7 A. M. At noon, on my second visit he was free from pain, and so continued.

In another case of a gentleman, age about 67, the attack was more severe than in the one mentioned. The muscles involved were those in the left illiac, running back to the lumbar, in the muscles of the urethra, neck of the bladder and rectum, giving rise to a constant feeling of tenesmus. The pain was extremely severe and exhausting. It had seized him between 4 and 5 A. M. I gave him about 7 grains of calomel, and told his wife that he would be well in an hour. This seems a long time to one panting under pain. In an hour, almost to the minute, the pain ceased suddenly, and did not return. I have seen cases of less severity, and always relieved them with calomel. The explanation of its action in such cases I conceive to be its property of restoring the damaged function of the liver from which the muscular rheumatism arises.

A remedy possessing such a wide range of action and power over morbid action of a beneficial chacracter has, nevertheless, fallen into disrepute and to a great extent, consequently, into disuse; but for what reason, it is needless now to inquire. What influence Homœopathy has had in bringing about this loss of deserved reputation may be greater than will be willingly recognized; yet it is a singular coincidence that the "regular" school has approached so closely the "irregular," as to be virtually practiced on the same principle, viz : symptomatology, a plan of prescribing for symptoms without reference to their cause, and seeks special remedies for special or particular symptoms. To regard symptoms as but indices of morbid action obliges the observer to look for their cause, and to attack it with his remedies, and not meet them as they are seen in whole or in part, as does the Homœopathist; as if there is pain to employ anodynes; if diarrhœa, astringents, or fever, antipyretics. What a remedy can do beyond allaying a symptom is not a matter of investigation except in a few isolated cases; yet the physiological action of a medicine is an index to its pathological use, and the pathological use discovers its influence or control over morbid action; and in this way leads to a rational mode of prescribing.

Had the influence of mercury over morbid action of a general character not been at first overlooked and subsequently fallen into oblivion, there would be no need or occasion now of referring to the subject. But from what appears now and then one might suppose that the value of mercury as a remedial agent is of but recent discovery. Of late, it has been ascertained that calomel is a *diuretic;* and by another *authority*, it has been declared to be useful in phthisis.* And so we may expect to find before the century closes, that it is an excellent remedy in many other forms or kinds of morbid action. Yet, I imagine, had its properties been known—properties once familiar to the general practitioner as a household word—its value as a diuretic or excellence in phthisis would not have a revelation of the decade of '80. So marked is its influence over morbid action, and so readily are its indications regarded, that its loss of reputation appears as one of inexplicable phenomena sometimes witnessed in the history of medicine.

A medicine, while affecting one organ more or less directly, may indirectly or through media, affect another and distant one, because the function of the latter is affected by a disturbed function of the former. It is not an unusual thing to see the excretion of the kidney altered in quantity and quality by a functional derangement of the liver and the kidney returning to its normal condition when the function of the liver has been restored; but in such a case, calomel can not be properly classed as a diuretic, where action is regarded as being the more immediately operative on the kidney. In phthisis, its action is indirect, by correcting conditions which act unfavorably on the morbid

---

*See Am. Jour. Med. Sciences, Dec., 88. p. p. 614-623.

action going on in the lung, as when digestion is impaired the powers of life are depressed, and this depression and its consequinces react on the disease in the lung. These facts I have observed for more than thirty years. In like manner, calomel has an influence over functional disturbances of the heart, every physician has oftentimes observed, because the heart symptoms arise from hepatic disturbance.

As a functionally disturbed action of the liver is attended by, or gives rise to morbid or abnormal action in other organs, it becomes of practical importance to note the evidences of such derangement. These are not difficult to observe, and they are so characteristic as to be well nigh pathognomonic.

First among these evidences is a characteristic pulse. This characteristic is incompressibility in different degrees, associated with this there is generally considerable fullness, though this is sometimes very slight. In a full pulse, the artery is large and carries a full stream. To have a name for this pulse, I call it a bilious pulse, because it is characteristic of biliousness.

In the next place, there is generally a furred tongue and if the hepatic derangement be chronic, there are oftentimes large sulci on the surface of the tongue. This is not unfrequently free of fur, or clean as fresh cut beef. In those who have suffered for a considerable time from hepatic indigestion, there is a congested state of the pharyngeal membrane; small blood vessels are seen on the surface of the membrane; also sulci and patches of mucous glands. These conditions are all heightened in those who use tobacco or intoxicants.

If the case be a chronic one, there is generally tenderness on percussion of the liver, in a greater or less degree. When this is present we can readily perceive the connection between a bilious pulse and an abnormal condition. We cup to relieve the tenderness, and when the cupping is completed we find the pulse small and soft and perfectly compressible. Nothing has intervened between the two conditions of the pulse but the cupping, and the difference we must ascribe to the action of the remedy. Is this but a simple and temporary depression of the heart's action produced by the impression made on it through the nervous system, or an actual relief of the liver? To this question we have a satisfactory answer given by another fact. This fact is that calomel has the same effect on a full and incompressible pulse, when we have the characteristic pulse, but no tenderness of the liver on percussion, the administration of calomel is followed by the like kind of pulse as that which followed cupping so that we know that cupping and calomel, affecting the pulse in the same way, have a like effect on the liver. Hence a bilious pulse is the evidence that calomel is indicated, it matters not with what kind of morbid action it may be connected, or from what arise. It will be found to change the full and incompressible pulse to one that is soft, small and compressible, and when the remedy has done this all fever, if present at any time of the attack, will be generally found to have abated and disappeared at the same time. If not, the further use of the remedy is still indicated.

Not only does the pulse become normal under the use of calomel, but all symptoms, or conditions of the system, dependent on the abnormal state of the liver, will disappear at the same time. Thus the tongue will clean, the pharyngeal membrane lose its conjested character, and assume the condition it was in before the attack.

It is sometimes the case that the remedy ceases to have any further apparent effect on the pulse or tongue ; some fever still continues, and skin a little dry, sometimes there is a slight but persistent cough, dry and hacking, when any of these symptoms still continue after the use of mercury for days or possibly for a week or two, the fact shows that the irritated liver does not wholly yield to the action of the remedy. In such cases, a blister over the liver will complete the cure ; the tongue cleans, the liver cleans, the skin becomes soft and velvety, and all fever disappears. In most instances, however, calomel effects the cure witout a blister. If the blister applied under such circumstances, is followed by a disappearance of all morbid symptoms, it cannot be doubted, when the same follows the use of calomel, that this remedy acts on the liver as readily, but differently, as does a blister.

Of the salts of mercury no one is equal in inflammatory affections and hepatic derangement, to the mild chloride. It is to be given for effect, and not in any uniform dose or with any fixed frequency. The effect to be looked for is that seen in the pulse and other concomitant symptoms. In ordinary cases of hapetic derangement, one grain in pill form will be found to be sufficient, given at night, and every night until the symptoms for which it was given have disappeared ; for these disappearing, we know their cause has been removed. But experience in the use of the remedy will soon enable one to adjust the dose and frequency to each particular case ; and when he has done so for a time, and observed closely the wide range of its action, he will find that he has in calomel a remedy whose influence over morbid action is without an equal in all cases in which its use is indicated.

